# Updating the Species Inventory of Large- and Medium-Sized Mammals in China Based on 2009–2020 Field Observation Records

**DOI:** 10.3390/ani14233380

**Published:** 2024-11-24

**Authors:** Shuyi Zhu, Jia Tian, Jianbing Yue, Fei Duan, Sheng Li

**Affiliations:** 1 Wildlife Conservation Monitoring Center, National Forestry and Grassland Administration, Beijing 100013, China; 19801308351@163.com (S.Z.); yuejianbing2008@sina.com (J.Y.); 2 School of Life Sciences, Institute of Ecology, Peking University, Beijing 100871, China; jia_tian@pku.edu.cn (J.T.); duanfei0318@163.com (F.D.); 3 Natural History Museum of China, Beijing 100050, China

**Keywords:** mammalian diversity, biodiversity inventory, species richness, national species list, camera-trapping, China

## Abstract

Large- and medium-sized mammals (LMMs) are closely related to humans and perform critical functions in the ecosystem. China harbors one of the richest mammalian faunas in the world, whereas there is an urgent need among wildlife managers and researchers to update the inventory of China’s LMM species based on recent empirical information. In this study, we compiled the occurrence records of terrestrial LMMs in China during 2009–2020, mainly from camera-trapping surveys, through a comprehensive literature search and systematical data collection. With a total of 42,937 occurrence records (12,397 after removing duplicates) from 5461 sites, we recorded 134 LMM species belonging to 8 orders and 23 families. Compared to the national species list, seven species are missing from field observation records, including four of Carnivora, two of Primates, and one of Lagomorpha. Yunnan is the province with the highest richness of recorded LMMs (n = 69), and Xizang is the only province where all seven large apex predators have been recorded. This study systematically updates the species inventory of LMMs in China based on observation records and will provide an important baseline to support and guide future studies, monitoring, and conservation management of this important functional group in China.

## Simple Summary

In this study, we systematically collected and summarized field observation records, mainly from camera-trapping surveys, during 2009–2020 for large- and medium-sized mammals (LMMs) in China. With an extensive dataset of 12,397 occurrence records from 5461 sites, we updated the species inventory of China’s LMMs, resulting in a total of 134 LMM species recorded. We summarized the richness of LMMs in each regional zone and province and examined the situation of the seven missing species compared to the national species list. This study systematically updates the status of LMM species in China and provides a reliable baseline for future study and conservation of this important functional group.

## 1. Introduction

Large- and medium-sized mammals (LMMs) are key functional groups in terrestrial ecosystems and play critical roles in shaping the ecosystem’s structure and dynamics [1]. Large carnivores sit at the top of the food chains and play a key role in maintaining stable populations of prey species through direct predation and indirect fear effect, which in turn help maintain the ecosystem composition and structure [1,2]. Large- and medium-sized herbivores can influence the composition and structure of plant communities through grazing and trampling [3] and indirectly influence various ecological processes such as carbon cycling and nitrogen mineralization [3,4]. Large- and medium-sized omnivores have specific ecological functions with energy and biomass flowing between multi-trophic species and may play important roles in seed dispersal, carcass scavenging etc. [5]. LMMs are typically characterized by a large home range, long life span, low density, slow regeneration, and small litter size [6,7]. Like most of the Earth’s large fauna, LMMs are the most closely related and iconic animals to humans. However, following the rapid economic development of human societies, LMMs have experienced severe population declines and range contractions globally over the past few centuries due to various pressures such as hunting, habitat loss, and land use change [8], resulting in a rather high threatened level among all terrestrial taxa [9,10].

As one of the world’s 17 mega-diversity countries [11], China harbors one of the richest mammalian faunas in the world [12]. In the context of the global biodiversity crisis, China is also experiencing severe biodiversity loss, especially for LMMs [2,13]. Przewalski’s horse (*Equus ferus*) and Père David’s deer (*Elaphurus davidianus*) have become extinct in the wild [14,15]. Several species, such as the Sumatran rhinoceros (*Dicerorhinus sumatraensis*), hog deer (*Axis porcinus*), and saiga (*Saiga tatarica*) that used to be distributed in China completely disappeared from the country during the 20th century (i.e., regional extinction) [12,16,17]. Many other species, such as the Chinese pangolin (*Manis pentadactyla*) and clouded leopard (*Neofelis nebulosa*), have experienced dramatic range shrinkage since the mid-20th century [18,19]. A wide retreat of large carnivores, including leopards (*Panthera pardus*), snow leopards (*P. uncia*), wolves (*Canis lupus*), and dhole (*Cuon alpinus*), has been reported across the vast area of Southwest China, and these apex predators are currently only occurring in a handful of reserves, as demonstrated by recent camera-trapping records [20].

On the other hand, in the past two decades, with the continuous growth of protected areas and increased efforts devoted to conservation, China has made remarkable achievements in improving its ecological environments [21,22]. The threatened status of many iconic species, such as giant panda (*Ailuropoda melanoleuca*), Przewalski’s gazelle (*Procapra przewalskii*), Tibetan antelope (*Pantholops hodgsonii*), Amur tiger (*Panthera tigris altaica*), Amur leopard (*P. pardus orientalis*), and Asian elephant (*Elephas maximus*), has improved with considerable population increase or recovery [23,24,25,26,27]. The reintroduction and rewilding of Przewalski’s horse, Père David’s deer, and giant panda have achieved impressive results [15,28,29]. Meanwhile, several new LMM species are discovered and described within China, including white-cheeked macaque (*Macaca leucogenys*) and Gaoligong Hoolock gibbon (*Hoolock tianxing*) [30,31], and new distribution records of LMM species recorded in China for its first time, such as Myanmar snub-nosed monkey (*Rhinopithecus strykeri*), golden jackal (*Canis aureus*), and red serow (*Capricornis rubidus*), have been increasingly reported [32,33,34]. Therefore, to better understand the distribution status of LMMs within the country, an urgent need has emerged among wildlife managers and researchers to update the inventory of China’s LMM species based on recent empirical information.

Reliable species inventory requires long-term monitoring efforts at a large scale and with extensive data accumulation. China has carried out two national surveys on its terrestrial vertebrates since 1990 [35,36]. The survey methodology relies mainly on traditional methods like line transect, and the accuracy of data depends largely on the ability and experience of the surveyors [37,38]. Given the considerable uncertainty of records collected in these national surveys, it is difficult to generate a reliable baseline of wildlife resources and then further determine its dynamics in a timely and effective manner [37]. Only for a handful of iconic flagship species, such as the giant panda, China has conducted well-designed regular surveys to determine their distribution and population changes, but this would be costly and not applicable to all species. In the past 20 years, camera trapping has been rapidly developed and widely applied to LMM surveys and studies in China [39]. It is estimated that by 2020, the ten major camera-trapping monitoring networks in China have accumulated tremendous data of more than 11 million photographs and videos from a total sampling effort of more than 7.5 million camera days [39]. The rapidly increasing publications based on camera-trapping surveys provide a large amount of empirical data and information on species occurrences, which could greatly contribute to species inventory and biodiversity research [39,40,41,42]. For example, as of 2019, a total of 165 mammal species belonging to 11 orders and 32 families have been recorded by the ten major camera-trapping monitoring networks in China, accounting for more than 23% of the total number of wild mammal species of the country [39]. Meanwhile, mammal studies using modern non-invasive DNA sampling and analysis techniques have also provided us with reliable distribution records of numerous elusive mammals in many regions of China in recent years [43,44,45]. In addition, the rapid development of citizen science and social media also provides us with a large number of sightings and photographic records [46], which has become a powerful supplement to determine the distribution status of specific species [18,47]. Integrating these field observation records from various sources will provide us with a sound data foundation to support the species inventory of LMMs in China.

In this study, we conducted a comprehensive literature search and systematically collected the occurrence records of LMMs generated from field observatories, mainly from camera-trapping surveys, from 2009 to 2020 in China. We summarized the current status of all terrestrial LMM species in China and examined the situation of “missing” species compared to the formal national species list. The results will provide us with an up-to-date inventory of LMMs in China and important baseline information to guide future studies, monitoring, and conservation management targeting these species.

## 2. Materials and Methods

### 2.1. Study Area

**Figure 1:**
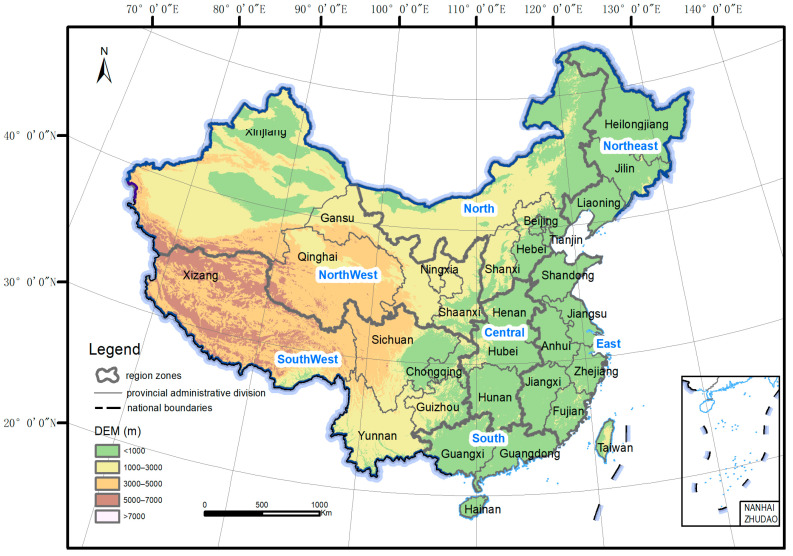
The topography, regional zones, and provinces of China.

We consider the entire territory of the People’s Republic of China, including the two large islands of Taiwan and Hainan, as our study area (Figure 1). Spanning a vast area from Central to East Asia, China has complex and diverse topography, including plateaus, plains, mountains, hills and basins. The elevation of China is, in general, high in the west and low in the east, with altitudes ranging from −155 to 8849 m a.s.l. (Figure 1).

Owing to its broad latitude and altitude ranges and the influence of East Asian monsoon, China has a wide climate spectrum, including cold temperate in the north, warm tropical in the south, arid continental in the northwest, and alpine on the west plateaus. These climates create various terrestrial ecosystems harboring highly diverse flora and fauna across the country [48,49]. There are 34 provincial-level administrative regions (hereafter referred to as provinces) in China, which are usually divided into seven regional zones: Northeast, North, Central, East, South, Southwest, and Northwest (Figure 1, Table 1).

### 2.2. Study Species

In this study, we used *Taxonomy and Distribution of Mammals in China* [12] as the taxonomic reference and considered its species list as the national list of China, which documents 694 species belonging to 12 orders and 58 families. There are no common criteria regarding the definition of LMMs, as researchers have used different cutoffs of the species’ body mass in different areas and contents of previous studies. In this study, we set the body mass as ≥1 kg for defining LMMs, a threshold that has been widely used in many previous studies [21,50,51]. The selection of target species followed three criteria:

(1) Body mass: adult body mass ≥ 1 kg;
(2) Habitat: only included species inhabiting terrestrial habitats. Marine species were excluded. Semi-aquatic species such as Eurasian otter (*Lutra lutra*) and Eurasian beaver (*Castor fiber*), as well as arboreal and semi-arboreal species (e.g., Primate species), were included;
(3) Excluding rodent families Sciuridae (squirrels and the relatives) and Spalacidae (moles and bamboo rats), although some species fulfill the first two criteria. As our data collection was mainly based on a camera-trapping survey, where the species identification is largely dependent on photos, it is difficult to correctly identify specific species solely based on external morphology. Meanwhile, the motion-triggered cameras have a rather low detection rate on these species, so we excluded them from this study.

We considered all terrestrial LMM species in the national list that meet these three criteria as our target species (Table S1).

### 2.3. Data Sources and Processing

We conducted a comprehensive literature search and review of the occurrence records and information of our target species from multiple sources within China from 2009 to 2020. Only the records generated from field observations were included, and the recording methods include camera-trapping, direct observation or capture, radio telemetry, and DNA molecular identification. Among them, camera trapping is considered one of the most effective methods for monitoring terrestrial LMMs and ground-dwelling birds [39,41,52]. Therefore, we primarily focused on the distribution records derived from camera-trapping studies and took those records with confirmed evidence from other sources as supplementary. The data sources include:

(a) Academic papers and theses based on camera-trapping surveys: We conducted a comprehensive literature search on published camera-trapping studies. We searched in Web of Science, Google Scholar, and China National Knowledge Infrastructure using “camera-trapping”, “camera trap”, and “China” as keywords and extracted the species list from each study. A total of 408 articles and theses were collected (see full list of the searched articles in online shared files).
(b) Other academic papers: For species that are difficult to document or have low detection rates in general camera-trapping surveys, such as arboreal primates (e.g., Hylobatidae), ungulates inhabiting open habitats (e.g., Przewalski’s gazelle), semi-aquatic otters and beavers and those species with less than 20 records from step (a), we used their species names (Chinese name, English name and scientific name) as keywords to conduct a species-specific literature search. A total of 241 articles with confirmed field observation records (e.g., direct sightings, carcasses, DNA samples, etc.) of these species were added to our dataset (see full list of the searched articles in online shared files).
(c) GBIF (Global Biodiversity Information Facility) database: We searched for LMM records of China in the GBIF database (https://www.gbif.org/ (accessed on 9 August 2023)) using the “rgbif” package in R 4.0.2 [53], resulting in 3002 records (GBIF.org GBIF Occurrence Download https://doi.org/10.15468/dl.e7uztx, accessed on 9 August 2023).
(d) News: It may take a long time for many camera-trapping studies to be published, whereas the detections of specific species, especially the rare ones, are usually reported through various media prior to the production of academic articles. Therefore, using “camera-trapping”, “camera traps”, and species names as keywords, we searched in Baidu News (https://news.baidu.com/ (accessed on 4 January 2021)), Bing (https://cn.bing.com/ (accessed on 4 January 2021)), and WeChat for news and media reports related to our target species. Sporadic records reported in posters and presentations during academic conferences were also collected and included. After examining the empirical evidence (e.g., photographs or videos of the reported animals), we collected 661 occurrence records from these sources with confirmed species information.
(e) Long-term monitoring data maintained by the authors: Our *Wildlife Ecology and Conservation* research group at Peking University has established a large regional camera-trapping network (the *Camera-trapping Network of the Mountains of Southwest China*) in Southwest China since 2002 [54]. In the past two decades, this network has accumulated a big dataset and the occurrence records of all LMM species were included in this study.
(f) Other unpublished data: We collected over 30,000 unpublished camera-trapping records from our collaborators, partners, and conservation NGOs such as the Chinese Felid Conservation Alliance (CFCA), Wilderness Xinjiang, and Qianjiangyuan National Park. We also collected information from 8 camera-trapping survey reports and 49 questionnaires on protected area’s camera-trapping survey results [39,55].

All literature and records were carefully examined by the authors and those without direct evidence or not being first-hand information were excluded from further analysis. We corrected all synonyms according to the *Taxonomy and Distribution of Mammals in China* [12] to avoid taxonomic confusion. We removed duplicate records and the records without clear geographic information. We extracted the geographic location of each occurrence site. If precise coordinates were available for a given record, we directly extracted the latitude and longitude of the location; if not, we queried the latitude and longitude coordinates of the center of the smallest land unit (e.g., protected areas, parks, timberlands, villages, etc.) using Baidu Maps or Google Earth. Vector layers of points were then generated in ArcGIS 10.5 (ESRI Inc., Redlands, CA, USA) as the occurrence sites for all target LMM species.

### 2.4. Data Statistics

To determine the threatened and protection status of each species, we examined their national Red List categories according to *China’s Red List of Biodiversity* [56] and national protection levels according to the *List of National Key Protected Wildlife* issued by the National Forestry and Grassland Administration (NFGA) and the Ministry of Agriculture and Rural Development (MARD) [57]. We are aware of the complicated taxonomy of several taxa, including *Budorcas*, *Ovis* species, and *Cervus elaphus*, for which *China’s Red List of Biodiversity* and *List of National Key Protected Wildlife* adopt alternative taxonomic opinions differing from the national list [12]. For example, the *List of National Key Protected Wildlife* recognizes four distinct *Budorcas* species, whereas the national list combines them into two species (i.e., Chinese takin *B. tibetana* and Himalayan takin *B. taxicolor*). The national list also considers all red deer clades (e.g., Tarim red deer *yarkandensi*, Tibetan red deer *wallichii*, and wapiti *canadensis,* etc.) in China as a single species, *Cervus elaphus*, and all argali clades, such as Kazakh argali *collium*, Marco Polo argali *polii,* and North China argali *jubata*, as a single species *Ovis ammon*. In this study, we assigned the highest protection level of the taxon recognized in the *List of National Key Protected Wildlife* to each species of these taxa (takin, red deer, and argali) in the national list.

We compiled the occurrence records for each regional zone and province, respectively. To understand the composition of LMMs in different regions, we counted the number of large apex predators (i.e., large carnivores that act as apex predators in the ecosystems where they inhabit) recorded in each regional zone and province. We considered the presence of large apex predators as an indicator of trophic completeness of the LMM community within the region [1,45]. In addition, we also do the same statistics for megafauna (≥44 kg) given their critical role in the ecosystem [58,59]. Following the definition of large carnivores proposed by Ripple et al. [2], we identified seven large apex predators in China, including tiger, leopard, snow leopard, clouded leopard, Eurasian lynx (*Lynx lynx*), wolf, and dhole. We excluded bear species (e.g., brown bear *Ursus arctos* and Asiatic black bear *U. thibetanus*) here, although they belong to the order Carnivora and are considered to be large carnivores in some studies (e.g., Ripple et al. [2]) since these species typically have an omnivorous dietary composition.

We searched and reviewed the historical literature of species with no confirmed observation records in China from 2009 to 2020 and collected the range maps or distribution records of these species from the IUCN Red List and *China’s Red List of Biodiversity* in order to sort out the distributional information of these “missing” species and discuss the potential reasons why they are not recorded.

## 3. Results

### 3.1. Data Summary

**Figure 2:**
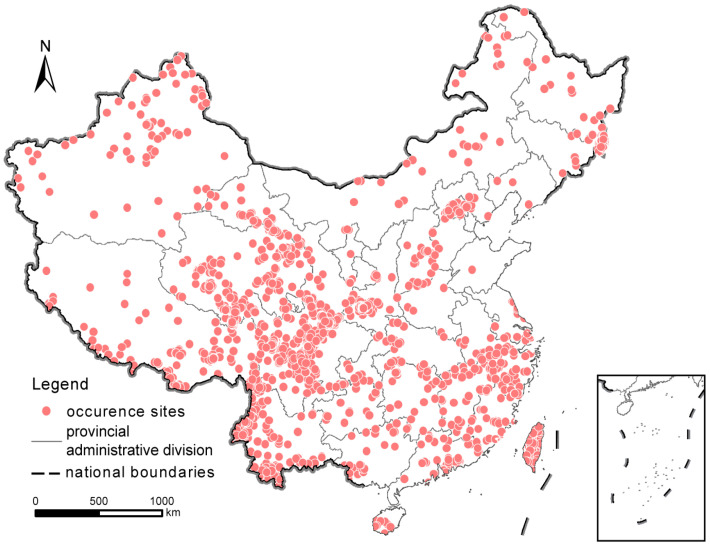
Occurrence sites of large- and medium-sized mammals recorded in China, 2009–2020. Number of sites n = 5461.

We extracted 42,937 raw occurrence records of LMMs from 5461 sites and 12,397 after data cleaning and removal of duplicates based on our data collection (Figure 2).

With respect to the records of individual provinces, Sichuan (27,434), Shaanxi (5479), and Taiwan (1815) (Table 1) were the three provinces with the highest numbers of raw data records, and Taiwan (1500), Sichuan (1329) and Shaanxi (454) were the three provinces with the highest numbers of records after data cleaning. Ningxia, Liaoning, Tianjin, and Shandong have less than ten occurrence sites (Figure 2, Table 1).

### 3.2. Species Richness

Based on the national list, we identified 141 LMMs belonging to 8 orders and 23 families as potential target species following our species selection criteria (Table S1). From the occurrence records we collected, 134 LMM species belonging to 8 orders and 23 families were recorded in China from 2009 to 2020, accounting for 95.04% of the national list, with no new ones beyond the list. Among them, three species (i.e., Przewalski’s horse, Père David’s deer, and saiga) were once extinct in China but are now back in the wild, benefiting from the establishment of reintroduced populations in the past two decades.

**Figure 3:**
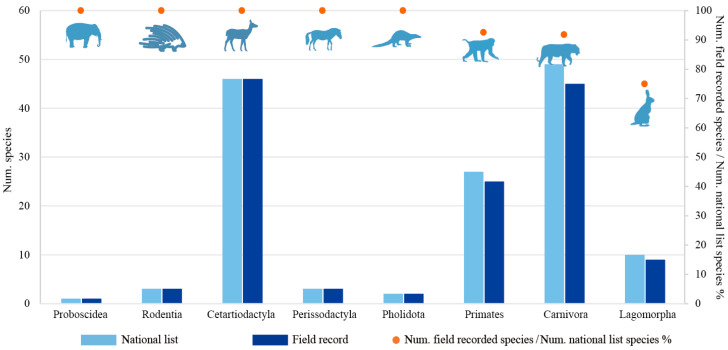
Number of large- and medium-sized mammal species in each order derived from national list (light blue) and field records (blue).

At the order level, Cetartiodactyla (46 species), Carnivora (45 species), and Primates (25 species) were the three orders with the highest richness (Figure 3). All species in Cetartiodactyla, Pholidota, Rodentia, Perissodactyla, and Proboscidea were recorded, whereas species in Carnivora (4 species missing), Primates (2 species missing), and Lagomorpha (1 species missing) were not fully recorded. These seven LMM species were included in the national list but not found in confirmed field records (Table 2). At the family level, the species of 17 families have been fully recorded in the field, with Hylobatidae (gibbons) being the family with the highest proportion of missing species (28.57%) (Figure S1).

Among the 134 recorded LMM species, 71 are listed as the first level of National Key Protected Wildlife, such as tiger, leopard, snow leopard, and Przewalski’s gazelle, and 43 as the second level, such as Pallas’s cat (*Otocolobus manul*), Asiatic black bear and blue sheep (*Pseudois nayaur*). There are 96 species being assessed as threatened, i.e., Critically Endangered CR (41 species), Endangered EN (33 species), or Vulnerable VU (22 species), in the Red List of China (Table S1).

### 3.3. Community Composition

Among the seven regional zones of China, the Southwest China region had the highest species richness (n = 102), accounting for 76.12% of all recorded species, whereas the Northeast region had the lowest richness (n = 25). The Southwest region also recorded the highest number of nationally protected species (first level n = 52, second level n = 33), while the Central region recorded the fewest (first level n = 7, second level n = 12). Megafauna species (body mass ≥ 44 kg) were recorded in all regions, but no large apex predators were recorded in the East and South China regions (Table 1).

**Figure 4:**
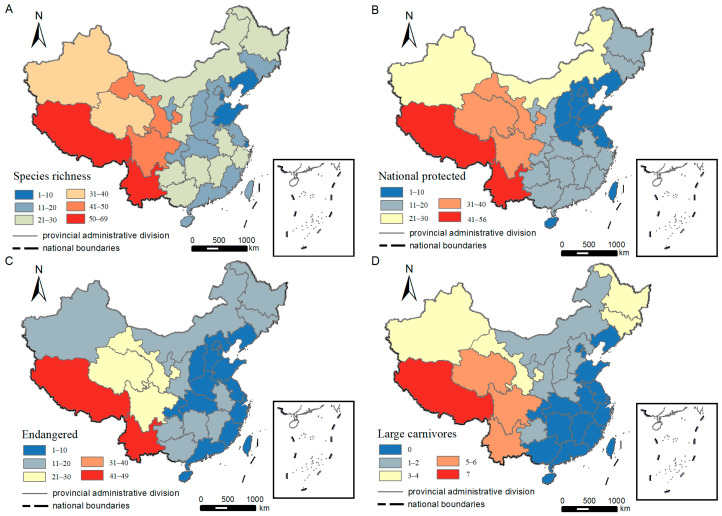
The large- and medium-sized mammals recorded in each province 2009–2020, China. (**A**)—Species richness (total number of LMM species recorded), (**B**)—Number of national key protected species recorded, (**C**)—Number of threatened species recorded, (**D**)—number of large apex predator species recorded.

In terms of different provinces, Yunnan (n = 69), Xizang (n = 64), Gansu (n = 49) and Sichuan (n = 46) recorded more than 40 LMM species, and Liaoning (n = 7), Tianjin (n = 7), Shanghai (n = 5) and Shandong (n = 2) recorded less than ten species (Table 1, Figure 4A). Yunnan (n = 31) recorded more than 30 first-level national key protected species (Table 1, Figure 4B), and Yunnan (n = 49) and Xizang (n = 46) recorded more than 40 threatened species (Table 1, Figure 4C). Fifteen provinces recorded at least one species of large apex predator, and Xizang was the only province that recorded all seven predators. No megafauna species was recorded in Shandong, Shanghai and Tianjin (Table 1, Figure 4D).

### 3.4. Overview of “Missing” Species

We examined the situation of the seven species with no confirmed records from 2009 to 2020 in China.

#### 3.4.1. White-Handed Gibbon (*Hylobates lar*)

Fan considered the white-handed gibbon to be extinct in China in his review of the population and conservation status of China’s gibbon species [31]. First discovered in China in 1964, the white-handed gibbons were native to Yunnan’s Menglian and Cangyuan but have been a rare species ever since (Figure S2B) [31,60,61]. Only 19–27 remaining individuals were reported in a 1985 survey, and an estimated 30–40 individuals in 1988 [61]. Despite numerous field surveys at possible distribution sites such as the Nangunhe Reserve and Menglian County in the past two decades, and although several nature reserves have been established since the 1980s to conserve this endangered small ape, no confirmed records have yet been reported [31,62]. This suggests that they may have gone extinct in China. Currently, the white-handed gibbons are still inhabiting the tropical rainforests of several Southeast Asia countries, including Indonesia (Sumatra), the Lao People’s Democratic Republic, Malaysia (Peninsular Malaysia), Myanmar, and Thailand [63]. The IUCN range map shows that the species distribution in China is located only in the Lincang region of Yunnan Province, a small part of the area close to Myanmar, and is categorized as Possibly Extinct [63] (Figure S2A).

#### 3.4.2. Northern White-Cheeked Gibbon (*Nomascus leucogenys*)

The northern white-cheeked gibbon is assessed as CR by the IUCN Red List, with wild remnant populations only occurring in Laos and Vietnam, and the vicinity of the Xishuangbanna Nature Reserve at southern Yunnan Province in China is categorized as Possibly Extinct [64] (Figure S2A). This species was once widely distributed in Mengla, Jiangcheng, and Lvchun counties in Yunnan (Figure S2B), with an estimated population of more than 1000 individuals in the 1960s; however, followed by a rapid decline, the population was estimated to be fewer than 100 individuals in the 1980s [65]. In the 1990s, it was considered on the edge of extinction, with a rather small, isolated population probably remaining only in the Xishuangbanna [66,67]. In 2011, a comprehensive survey conducted at the Xishuangbanna Reserve found no signs of the existence of this species, so it is thought to have disappeared or become functionally extinct in China [68]. Currently, a small semi-captive population is rising at the Wild Elephant Valley in Xishuangbanna, which may serve as a foundation for future reintroduction projects of this species [66,69].

#### 3.4.3. Bengal Fox (*Vulpes bengalensis*)

Bengal fox is endemic to the Indian subcontinent [70]. It is suspected that this fox may occur in the lowland foothills of the East Himalayas in China (i.e., southeastern Xizang), but there are no confirmed records in the past two decades.

#### 3.4.4. Sloth Bear (*Melursus ursinus*)

The sloth bear is mainly distributed from the southern foothill of the Himalayas to the central and southern Indian subcontinent and Sri Lanka [71]. It is suspected that this bear may occur in southeastern Xizang following some historical but maybe unreliable records, yet there are no confirmed field records reported.

#### 3.4.5. Smooth-Coated Otter (*Lutrogale perspicillata*)

As the largest of China’s three otter species that have all experienced dramatic population declines and range contractions due to direct harvesting in history [72,73,74], the smooth-coated otter historically inhabits rice paddies and forested waterfronts [75]. Historical records of this species are from the Pearl River Delta in South China, the Lancang River (i.e., the Mekong), Red River and Dulong River (i.e., the Irrawaddy) in Yunnan of Southwest China, as well as the Yarlung Zangbo River (i.e., the Brahmaputra) in southeastern Xizang (Figure S2D) [75,76]. However, it has not been recorded in the wild throughout the country at least since 2006 [77]. The IUCN Red List considers the smooth-coated otters to be Possibly Extinct in China, with possible distribution in southwestern Yunnan [76] (Figure S2C).

#### 3.4.6. Jungle Cat (*Felis chaus*)

Although jungle cat has been long included in China’s mammal list, few records and specimens were collected in the country. We only found the following information: a 1917 specimen without coordinates in GBIF; a 1996 specimen from Shoulu Mountain Nature Reserve, Jingtai County, Gansu Province, which is now preserved in the East China Normal University but probably a wrongly identified record [78]; and coarse historical records included in *A Guide to The Mammals of China* (Figure S2F) [75]. The IUCN range map shows a relatively wide distribution in Xizang, Sichuan, and Yunnan (Figure S2E), but there are no confirmed records of this species in the past two decades despite extensive camera-trapping survey efforts across this region.

#### 3.4.7. Korean Hare (*Lepus coreanus*)

Both the IUCN Red List and China’s national list recognize the Korean hare as a distinct species [12,79], whereas its taxonomic status somehow remains disputed [56]. The IUCN range map shows that the Korean hare is primarily distributed across the Korean Peninsula, with a small part extended to southern Jilin Province in China [79] (Figure S2G). The geographic division between the Korean hare and its close relative Manchurian hare (*L. mandshuricus*), which is mainly distributed in Northeast China and the Ussuri region of Russia [79], has not been well determined. The camera-trapping studies and other field surveys in Northeast China reported no confirmed records of Korean hare during recent years.

## 4. Discussion

### 4.1. Survey Effort and Coverage

Based on a large amount of empirical data and information, our study compiled the occurrence records and updated the species inventory of LMMs in China. The results provide reliable baseline information for future conservation and research on this important functional group. In the past two decades, China has conducted extensive wildlife field surveys and accumulated a large amount of field observation data. These records and information provide a sound basis for a comprehensive understanding of the status of LMMs across the country [39,41]. The records and data collected in this study also have great potential for examining the LMM’s spatial patterns of diversity and the underlying drivers. Taking advantage of increasingly accumulated occurrence records, recent studies have successfully generated high-resolution distribution maps of multiple individual species within the country, such as Asiatic black bears [80], Eurasian lynx [47], North China leopards (*P. pardus japonensis*) [81], and Chinese pangolin [18]. Future studies shall consider further data integration and mining to address the key scientific questions in LMMs diversity, and the results will have further implications to guide future survey efforts and policymaking in conservation management.

Although the occurrence sites of LMMs collected in this study have covered all provinces in China, the distribution of these sites is somehow spatially biased due to uneven sampling across the country. The existing occurrence sites are mainly concentrated in mountainous regions with high species richness, such as the Qilian Mountains, Hengduan Mountains, Qinling Mountains, Taihang Mountains, Changbai Mountains, and Wuyi Mountains, whereas there are fewer survey sites in the North China Plain, Northeast China Plain, central and western Inner Mongolia, and southern Xinjiang (Figure 2). If these records are used in future large-scale spatial analyses of biodiversity patterns, the gaps in these areas will become obvious sampling biases that have to be considered in the analysis and modeling. In the future, to effectively fill these gaps, a more comprehensive and systematic top-level design should be considered in the construction of regional and national wildlife monitoring networks so that the observation records can more accurately describe the spatial and temporal distribution of LMMs across the country.

Besides camera-trapping efforts, some other approaches, such as modern molecular analysis and citizen science, also have great potential to provide us with reliable occurrence data of LMM species and then improve the data coverage. For detecting elusive or rare species, we can consider using molecular technologies such as eDNA and iDNA through DNA meta-barcoding, which can supplement occurrence data of certain species or taxa and expand the scope of future research from simply inventory to inter-specific relationships and food web structure [44,82,83]. Citizen science is another promising method that can provide large amounts of data from more areas [84]. Citizen science has already made important contributions to bird surveys and research. With birdwatchers in China uploading their observation records to the online open platform China BirdReport (http://www.birdreport.cn/ (accessed on 30 September 2024)), it is estimated that there are currently more than 20,000 active users of the platform, resulting in over 1.21 million records of 1353 bird species across the country as of 2021 [85]. Although mammals are relatively difficult to observe compared to birds, multiple citizen science projects focusing on wild mammals have been proposed in China in recent years. For example, Wilderness Xinjiang (a conservation NGO) initiated the “Mammals Chasing” in 2014 [86], and Shanghai initiated the “Population survey of raccoon dogs (*Nyctereutes procyonoides*)” by volunteers in 2022 [87]. These projects provide opportunities for the public to participate in scientific surveys and research, which may serve as important sources continuously generating occurrence records of LMMs across a large scale.

### 4.2. Potential Reasons of Missing Species

Through systematic data collection, we did not find confirmed records of seven LMM species in the past two decades compared to the national list, which may be due to various reasons. One possible reason is incomplete data collection. Although we collected a large amount of survey data from various sources, it is difficult to ensure perfect comprehensiveness covering all published literature and grey literature. Moreover, many field surveys and datasets have not been published, and these records are difficult to reach through public access and literature platforms.

In addition, there are several other possible reasons: (1) Many areas in China still lack comprehensive field surveys (e.g., the eastern Himalayas) due to complex topography and poor accessibility. Therefore, it is difficult to verify the distribution status of species suspected to be occurring in these remote areas (e.g., Bengal foxes and sloth bears). (2) The taxonomic status of specific species (e.g., Korean hare) remains disputed, and it is difficult to morphologically distinguish them from other similar species in the field. (3) Some species may have disappeared from their historical regions or even the whole country (e.g., smooth-coated otter), but their regional extinction has not yet been confirmed by the national list. Determining whether a species is regionally extinct is often difficult, and the announcement is normally made upon extensive efforts of long-term investigations and serious assessments with great care, usually lagging many years behind the real elimination of the animals in the wild [88].

### 4.3. Species Richness Pattern

According to our results, Yunnan is the province with the highest LMM richness in China. Yunnan is in the southwestern part of China, characterized by low latitudes, broad elevation ranges, and highly diverse ecosystem types [89]. Special biogeographical location and complex topographic and climatic conditions provide important conditions for the rich fauna and flora in the region. ‘What determines species diversity?’ is one of the key questions to be addressed in ecological and biogeographical research [90]. It was found that climate stability, habitat complexity, and ecosystem type all play important roles in driving species richness [91,92,93]. Stevens suggested that under stable climatic conditions, species richness is high in the region [91], and Zhao et al. found that altitude difference played the most dominant role in mammal richness in their study of vertebrate richness in protected areas of China [94]. Chi et al. found that terrestrial mammal richness in China is closely related to forest ecosystems [93]. All these factors may contribute to the high richness of LMMs in Yunnan, whereas more in-depth studies and analyses are still needed. In this study, Shandong is found to be the province with the lowest LMM richness. The insufficient survey (few survey sites) in Shandong is one of the potential reasons behind this. In addition, Shandong is in the eastern part of China with a very high human population density and land use intensity and a very long history of human civilization and agriculture, which altogether has caused over-exploitation of natural resources with significant defaunation of LMMs as one consequence [95,96].

### 4.4. Community Integrity

In this study, we found that Xizang is the only province currently harboring all seven large apex predators. In about half of the rest provinces, at least one large apex predator has been recorded. However, no large apex predators have been recorded since 2009 throughout the entire East and South China regions. Sitting at the top of the food chain, large apex predators are able to regulate prey species through both direct pathways, such as predation, and indirect pathways, such as the fear effect, which in turn affects other trophic levels within the ecosystems through trophic cascades and therefore maintains the stability of ecosystem structure and function [1,2]. They are often considered the indicators of community integrity and ecosystem health [1,2]. Historically, all these seven large apex predators are widely distributed in China. For instance, leopards were once distributed in most provinces and regions of mainland China except for the arid lands and cold plateaus in the west [97]. Clouded leopards were once found in 17 provinces across the tropical and sub-tropical regions of the country [19], and wolves were still occurring in 29 mainland provinces in the mid-20th century [98]. However, the data we collected in this study showed that leopards are only recorded in 11 provinces, clouded leopards are currently only confirmed in small parts of Yunnan and Xizang, and wolves are currently recorded in only eight provinces. These results suggested that the ranges of most, if not all, large apex predators in China have dramatically shrunk, which is consistent with the general trend of large carnivores’ global decline [10]. Large apex predators are currently lacking in the entire East and South China, and much of Central China, and researchers and managers in these areas should pay attention to the ecological consequences following the elimination of these predators, such as herbivore overabundance, mesocarnivore release, and ecosystem trophic downgrading [99,100].

### 4.5. Future Outlook and Conservation Implications

Given the spatial distribution extent of camera-trapping efforts and LMM richness, China should improve the overall sampling design of LMM monitoring at the state level, where camera-trapping may serve as the primary survey tool, to gradually fill the monitoring gaps and reduce sampling bias [39,41]. The integration of citizen science approaches and the use of social media platforms to monitor wildlife will also provide us with important sources of observation data in the future [101]. Specific survey efforts shall be devoted to areas with few field studies, such as the provinces of Liaoning, Tianjin, Shandong, southern Xinjiang, and western Inner Mongolia. The data and results generated from these regional and national monitoring networks will serve well as the basis for future species inventory of the country. Promoting data sharing and data integration is another important issue [102]. Currently, due to various limitations of domestic policies and regulations, there are still many difficulties and challenges for Chinese wildlife researchers and managers while sharing biodiversity records with open-data platforms, especially international and abroad databases. In the future, the government, relevant agencies, and scientists shall work together to improve and promote the sharing of wildlife monitoring data and develop and establish data-sharing policies, protocols, and meta-data structures that could be commonly adopted by all parties. These efforts will provide strong support for regular updating of the country’s species inventory and in-depth studies on specific species or research topics [102]. Meanwhile, transboundary collaborations are urgently needed between China and neighboring countries to better examine the status of species with marginal distribution in China and to better protect their transboundary populations [19,103,104]. For species with no confirmed records and insufficient information, it is necessary to conduct specific research projects and field surveys to determine their distribution status within the country. For regions already with significant ecological consequences resulting from the elimination of large apex predators (e.g., overabundant herbivores such as wild boar *Sus scrofa*), future conservation actions may consider population control measures on the overabundant prey species and/or reintroduction of large carnivores or facilitating the natural dispersal of their existing source populations to restore the ecosystem’s trophic complexity [105,106].

## 5. Conclusions

In this study, we focused on the update of the species inventory of LMMs in China and showed the species richness pattern of LMMs at the province level. Through comprehensive literature search and systematical data collection, we found that 134 LMM species belonging to 8 orders and 23 families were recorded, mainly derived from camera-trapping surveys, from 2009 to 2020 across the country. Compared to the national species list, we identified seven species with no confirmed records in the field during the last decade and reviewed the historical literature of these missing species to discuss the potential reasons why they were not recorded. Furthermore, we summarized the richness of LMMs in each regional zone and province, and the results showed that Yunnan is the province with the highest richness of recorded LMMs (n = 69), and Xizang is the only province where all seven large apex predators have been recorded. Our results will provide an important baseline for future conservation of this important functional group in China.

## Figures and Tables

**Table 1:** Records of large- and medium-sized mammals in each province of China, 2009–2020.

Regional Zones	Provinces	No. Raw Data Records	Sites After Removing Duplicates	Species Recorded in the Field	Species at First National Protection Level	Species at Second National Protection Level	Threatened Species	Large Apex Predators	Mega Species
Northeast	Heilongjiang	107	23	22	8	10	14	4	8
	Jilin	195	96	20	6	10	14	3	7
	Liaoning	15	3	7	0	2	2	0	1
North	Beijing	222	29	13	2	5	4	0	3
	Hebei	186	51	14	2	5	5	1	3
	Inner Mongolia	134	34	27	8	14	16	2	8
	Shanxi	93	18	12	2	4	4	1	2
	Tianjin	19	1	7	0	2	2	0	0
East	Anhui	533	100	21	4	8	11	0	4
	Fujian	115	44	20	2	9	8	0	4
	Jiangsu	32	15	11	1	4	3	0	2
	Jiangxi	207	41	24	4	11	12	0	5
	Shandong	2	1	2	0	1	1	0	0
	Shanghai	18	14	5	0	2	1	0	0
	Taiwan	1815	1500	15	5	5	8	0	4
	Zhejiang	1595	283	21	4	9	10	0	4
South	Guangdong	186	58	19	2	9	8	0	4
	Guangxi	261	32	24	7	10	16	0	3
	Hainan	62	16	14	4	6	7	0	3
	Hongkong	497	422	10	2	3	4	0	1
	Macao	2	1	1	0	1	1	0	0
Central	Henan	59	11	18	2	8	7	1	3
	Hubei	160	22	19	4	8	9	0	5
	Hunan	157	41	23	4	10	11	0	4
Northwest	Gansu	544	49	49	18	21	28	4	18
	Ningxia	78	6	20	4	9	11	1	5
	Qinghai	563	263	38	14	18	23	5	12
	Shaanxi	5479	454	27	9	10	13	2	7
	Xinjiang	288	85	34	14	14	19	4	13
Southwest	Chongqing	61	10	17	4	7	8	0	3
	Guizhou	300	30	27	7	10	15	1	3
	Sichuan	27,434	1329	46	16	21	27	5	14
	Xizang	380	142	64	30	26	46	7	18
	Yunnan	1138	237	69	31	24	49	5	12

**Table 2:** The large- and medium-sized mammal species listed in the national list but with no confirmed records from 2009 to 2020 in China.

Species	Endemic to China	National Key Protected Level	China Red List Level	IUCN Red List Level
**I Primates**				
(1) Hylobatidae				
1. White-handed gibbon *Hylobates lar*	No	I	CR	EN
2. Northern white-cheeked gibbon *Nomascus leucogenys*	No	I	CR	CR
**II Carnivora**				
(2) Canidae				
3. Bengal fox *Vulpes bengalensis*	No		DD	LC
(3) Ursidae				
4. Sloth bear *Melursus ursinus*	No	II	EN	VU
(4) Mustelidae				
5. Smooth-coated otter *Lutrogale perspicillata*	No	II	CR	VU
(5) Felidae				
6. Jungle cat *Felis chaus*	No	I	CR	LC
**III Lagomorpha**				
(6) Leporidae				
7. Korean hare *Lepus coreanus*	No		LC	LC

## Data Availability

The species list included in the analysis and detailed information on the missing species are included in the Supplementary Materials. The dataset of occurrence sites used for this analysis is available in ResearchGate (https://doi.org/10.13140/RG.2.2.16321.57443). The sources of mammal records included in this study in China is available in ResearchGate (https://doi.org/10.13140/RG.2.2.36682.79041). For details about the dataset and other materials and information, please contact Shuyi Zhu and Sheng Li.

## Supplementary Materials

The following supporting information can be downloaded at: https://www.mdpi.com/article/10.3390/ani14233380/s1. Table S1: List of China’s 141 large- and medium-sized mammal species included in this study; Figure S1: The percentage of recorded large- and medium-sized mammal species compared to the national species list in each family; Figure S2. The distribution of large- and medium-sized mammals in China that are not recorded in IUCN and the China Species Red List.

## References

[1] Hoeks S., Huijbregts M.A.J., Busana M., Harfoot M., Svenning J.-C., Santini L., Santini L. (2020). Mechanistic insights into the role of large carnivores for ecosystem structure and functioning. Ecography.

[2] Ripple W.J., Estes J.A., Beschta R.L., Wilmers C.C., Ritchie E.G., Hebblewhite M., Berger J., Elmhagen B., Letnic M., Nelson M.P. (2014). Status and ecological effects of the world’s largest carnivores. Science.

[3] Forbes E.S., Cushman J.H., Burkepile D.E., Young T.P., Klope M., Young H.S. (2019). Synthesizing the effects of large, wild herbivore exclusion on ecosystem function. Funct. Ecol..

[4] McNaughton S., Ruess R.W., Seagle S.W. (1988). Large mammals and process dynamics in African ecosystems. BioScience.

[5] Kratina P., LeCraw R.M., Ingram T., Anholt B.R. (2012). Stability and persistence of food webs with omnivory: Is there a general pattern?. Ecosphere.

[6] Cardillo M., Mace G.M., Jones K.E., Bielby J., Bininda-Emonds O.R.P., Sechrest W., Orme C.D.L., Purvis A. (2005). Multiple causes of high extinction risk in large mammal species. Science.

[7] Chichorro F., Juslén A., Cardoso P. (2019). A Review of the relation between species traits and extinction risk. Biol. Conserv..

[8] Pacifici M., Rondinini C., Rhodes J.R., Burbidge A.A., Cristiano A., Watson J.E.M., Watson J.E.M., Woinarski J.C.Z., Di Marco M., Di Marco M. (2020). Global correlates of range contractions and expansions in terrestrial mammals. Nat. Commun..

[9] Ceballos G., Ehrlich P.R., Barnosky A.D., García A., Pringle R.M., Palmer T.M. (2015). Accelerated modern human–induced species losses: Entering the sixth mass extinction. Sci. Adv..

[10] Wolf C., Ripple W.J. (2017). Range contractions of the world’s large carnivores. R. Soc. Open Sci..

[11] Hilton-Taylor C. (2000). The 2000 IUCN Red List of Threatened Species.

[12] Wei F.W. (2022). Taxonomy and Distribution of Mammals in China.

[13] Mace G.M., Norris K., Fitter A. (2012). Biodiversity and ecosystem services: A multilayered relationship. Trends Ecol. Evol..

[14] King S.R.B., Boyd L., Zimmermann W., Kendall B.E. (2015). *Equus ferus* (errata version published in 2016). IUCN Red List. Threat. Species.

[15] Bai J.D., Zhang Y.Y., Zhong Z.Y., Cheng Z.B., Cao M., Meng Y.P. (2021). The 35th anniversary of the reintroduction of Milu deer to China: History, population status, achievements and challenges. Biodivers. Sci..

[16] Ding C., Liu J., Li C., Jiang Z. (2021). Probable extirpation of the hog deer from China: Implications for conservation. Oryx.

[17] IUCN SSC Antelope Specialist Group (2023). *Saiga* *tatarica*. IUCN Red List. Threat. Species.

[18] Kong Y.Q., Li S., Liu B.Q., Zhou J.J., Li C., Yu J.P. (2021). Distribution records and conservation status of Chinese pangolin (*Manis pentadactyla*) in China during 2010-2020. Biodivers. Sci..

[19] Ma Z., He Z., Wang Y., Song D., Xia F., Cui S., Su H., Deng J., Li P., Li S. (2022). An update on the current distribution and key habitats of the cloud leopard (*Neofelis nebulosa*) populations in China. Biodivers. Sci..

[20] Li S., McShea W.J., Wang D., Gu X., Zhang X., Zhang L., Shen X. (2020). Retreat of large carnivores across the giant panda distribution range. Nat. Ecol. Evol..

[21] Zhang H., Xu E. (2017). An evaluation of the ecological and environmental security on China’s terrestrial ecosystems. Sci. Rep..

[22] Li Y., Li Z.L., Wu H., Zhou C.H., Liu X.Y., Leng P., Yang P., Wu W.B., Tang R.L., Shang G.F. (2023). Biophysical impacts of earth greening can substantially mitigate regional land surface temperature warming. Nat. Commun..

[23] Buzzard P.J., Wong H.M., Zhang H. (2012). Population increase at a calving ground of the endangered Tibetan antelope *Pantholops hodgsonii* in Xinjiang, China. Oryx.

[24] Li C., Jiang Z., Ping X., Cai J., You Z., Li C., Wu Y. (2012). Current status and conservation of the endangered Przewalski’s gazelle *Procapra przewalskii*, endemic to the Qinghai–Tibetan Plateau, China. Oryx.

[25] Rozhnov V.V., Naidenko S.V., Hernandez-Blanco J.A., Chistopolova M.D., Sorokin P.A., Yachmennikova A.A., Blidchenko E.Y., Kalinin A.Y., Kastrikin V.A. (2021). Restoration of the Amur tiger (*Panthera tigris altaica*) population in the northwest of its distribution area. Biol. Bull..

[26] Wang T., Feng L., Mou P., Wu J., Smith J.L., Xiao W., Yang H., Dou H., Zhao X., Cheng Y. (2016). Amur tigers and leopards returning to China: Direct evidence and a landscape conservation plan. Landsc. Ecol..

[27] Wang Z.H., Chen F., Yang Z.C., Wang M.J. (2021). Research status and prospect of Asian elephant. For. Constr..

[28] Yang Z.S., Gu X.D., Nie Y.G., Huang F., Huang Y., Dai Q., Hu Y.B., Yang Y., Zhou X., Zhang H.M. (2018). Reintroduction of the giant panda into the wild: A good start suggests a bright future. Biol. Conserv..

[29] Zhang Y.Y., Bai J.D., Annah Z., Chen R.S., Xue D.Y., Zhong Z.Y., Cheng Z.B. (2021). Reversing extinction in China’s Père David’s deer. Science.

[30] Li C., Zhao C., Fan P.F. (2015). White-cheeked macaque (*Macaca leucogenys*): A new macaque species from Modog, southeastern Tibet. Am. J. Primatol..

[31] Fan P. (2017). The past, present, and future of gibbons in China. Biol. Conserv..

[32] Long Y., Momberg F., Ma J., Wang Y., Luo Y., Li H., Yang G., Li M. (2012). *Rhinopithecus strykeri* found in China!. Am. J. Primatol..

[33] Dong L., Luo H., Li S. (2019). Golden jackal (*Canis aureus*) recorded in Jilong County, Tibet. Acta Theriol. Sin..

[34] Chen X., Guan T., Jiang W., Li D., Yang K., Li S. (2021). Distribution and population status of bovine species in China based on bibliometric analysis. Biodivers. Sci..

[35] Ruan X.D. (1995). National terrestrial wildlife census and monitoring launched. Chin. J. Wildl..

[36] Gao E.H., Wang Z.C., Wang W.S., Chen D.F., Ma G.Q., Tang X.P. (2014). Technical plan for the second national survey of terrestrial wildlife in China. Chin. J. Wildl..

[37] Yu D.M., Deng S.Q., Liu Y.Z., Ruan X.D., Hu H.J. (2024). Experiences and prospects for national survey of terrestrial wildlife resources in China. Chin. J. Appl. Ecol..

[38] Li S., Mcshea W.J., Wang D., Huang J.Z., Shao L.K. (2012). A direct comparison of camera-trapping and sign transects for monitoring wildlife in the Wanglang National Nature Reserve, China. Wildl. Soc. Bull..

[39] Li S. (2020). Development progress and outlook of the wildlife camera-trapping networks in China. Biodivers. Sci..

[40] Li S., Wang D.J., Xiao Z.S., Li X.H., Feng L.M., Wang Y. (2014). Camera-trapping in wildlife research and conservation in China: Review and outlook. Biodivers. Sci..

[41] Xiao Z.S., Xiao W.H., Wang T.M., Li S., Lian X.M., Song D.Z., Deng X.Q., Zhou Q.H. (2022). Wildlife monitoring and research using camera-trapping technology across China: The current status and future issues. Biodivers. Sci..

[42] Xiao Z.S., Li X.Y., Quan R.C., Lian X.M., Li M., Nie Y.G., Xiang Z.F., Yang W.K., Xu F., Wang J. (2023). Construction of Sino BON mammal diversity monitoring network (Sino BON-Mammal): A 10-year review and future outlook. Biodivers. Sci..

[43] Shao X., Lu Q., Liu M., Xiong M., Bu H., Wang D., Liu S., Zhao J., Li S., Yao M. (2021). Generalist carnivores can be effective biodiversity samplers of terrestrial vertebrates. Front. Ecol. Environ..

[44] Shao X., Lu Q., Xiong M., Bu H., Shi X., Wang D., Zhao J., Li S., Yao M. (2021). Prey partitioning and livestock consumption in the world’s richest large carnivore assemblage. Curr. Biol..

[45] Lu Q., Cheng C., Xiao L., Li J., Li X., Zhao X., Lu Z., Zhao J., Yao M. (2023). Food webs reveal coexistence mechanisms and community organization in carnivores. Curr. Biol..

[46] Zhang J., Chen S.B., Chen B., Du Y.J., Huang X.L., Pan X.B., Zhang Q. (2013). Citizen science: Integrating scientific research, ecological conservation and public participation. Biodivers. Sci..

[47] Liu K., Liu Y., Li S. (2023). The current distribution and prediction of suitable habitat of Eurasian lynx (*Lynx lynx*) in China. Acta Theriol. Sin..

[48] Zhang W.P. (1998). Compilation of China’s biodiversity: A country study. China’s Biodiversity: A Country Study.

[49] Xu W.H., Ouyang Z.Y., Huang H., Wang X.K., Miao H., Zheng H. (2006). Priority analysis on conserving China’s terrestrial ecosystems. Acta Ecol. Sin..

[50] Bernardo P.V.D.S., Melo F.R.D. (2013). Assemblage of medium and large size mammals in an urban semideciduous seasonal forest fragment in Cerrado biome. Biota Neotrop..

[51] Huang G., Sreekar R., Velho N., Corlett R.T., Quan R.-C., Tomlinson K.W. (2020). Combining camera-trap surveys and hunter interviews to determine the status of mammals in protected rainforests and rubber plantations of Menglun, Xishuangbanna, SW China. Anim. Conserv..

[52] Zhu S.Y., Duan F., Li S. (2017). Promoting diversity inventory and monitoring of birds through the camera-trapping network in China: Status, challenges and future outlook. Biodivers. Sci..

[53] Chamberlain S.A., Boettiger C. (2017). R python, and Ruby clients for GBIF species occurrence data. PeerJ Prepr..

[54] Li S., McShea W.J., Wang D.J., Shen X.L., Bu H.L., Guan T.P., Wang F., Gu X.D., Zhang X.F., Liao H.H. (2020). Construction progress of the Camera-trapping Network for the Mountains of Southwest China. Biodivers. Sci..

[55] Tian J., Zhu S.Y., Zhang X.F., He L.W., Gu X.D., Guan T.P., Li C. (2021). The diversity of large- and medium-sized terrestrial mammals and birds in the Giant Panda National Park: A meta-analysis based on camera-trapping data. Biodivers. Sci..

[56] Jiang Z.G. (2021). China’s Red List of Biodiversity: Vertebrates.

[57] National Forestry and Grassland Administration (NFGA) and the Ministry of Agriculture and Rural Development (MARD) (2021). List of Wildlife Under National Key Protection. https://www.gov.cn/xinwen/2021-02/09/5586227/files/e007df5cdb364bcdbcb89d169047d6c5.pdf.

[58] Barnosky A.D. (2008). Megafauna biomass tradeoff as a driver of Quaternary and future extinctions. Proc. Natl. Acad. Sci. USA.

[59] Hansen D.M., Galetti M. (2009). The forgotten megafauna. Science.

[60] Yang D., Zhang J., Li C. (1987). Preliminary survey on the population and distribution of gibbons in Yunnan Province. Primates.

[61] Ma S.L., Wang Y.X. (1988). The recent distribution, status and conservation of Primates in China. Acta Theriol. Sin..

[62] Grueter C.C., Jiang X., Konrad R., Fan P., Guan Z., Geissmann T. (2009). Are *Hylobates lar* extirpated from China?. Int. J. Primatol..

[63] Brockelman W., Geissmann T. (2020). *Hylobates* *lar*. IUCN Red List. Threat. Species.

[64] Rawson B.M., Nguyen M.H., Coudrat C.N.Z., Roos C., Jiang X., Duckworth J.W. (2020). *Nomascus leucogenys* (errata version published in 2020). IUCN Red List. Threat. Specie.

[65] Hu Y., Xu H.W., Yang D.H. (1989). The studies on ecology in *Hylobates leucogenys*. Zool. Res..

[66] Fan P., Sheng H. (2009). The northern white-cheeked gibbon (*Nomascus leucogenys*) is on the edge of extinction in China. Gibbon J..

[67] Song Z.Y., Yang H.P., Yang Z.B., Yu D.L., Yang Z.C. (2017). Population status and conservation of *Nomascus leucogenys* in Xishuangbanna. J. West China For. Sci..

[68] Fan P., Fei H., Luo A. (2014). Ecological extinction of the critically endangered northern white-cheeked gibbon *Nomascus leucogenys* in China. Oryx.

[69] Li Z.Y., Xu D.Z., Gan Y.J. (2009). For the First Time, a White-Cheeked Gibbon Was Successfully Bred in the Wild. https://www.docin.com/p-516725029.html.

[70] Jhala Y. (2016). *Vulpes* *bengalensis*. IUCN Red List. Threat. Species.

[71] Dharaiya N., Bargali H.S., Sharp T. (2020). *Melursus ursinus* (amended version of 2016 assessment). IUCN Red List. Threat. Species.

[72] Zhang L., Wang Q., Yang L., Li F., Chan B.P., Xiao Z., Li S., Song D., Piao Z., Fan P. (2018). The neglected otters in China: Distribution change in the past 400 years and current conservation status. Biol. Conserv..

[73] Xu L.H. (1984). Otter species and resource conservation in China. Wild Anim..

[74] Zhang L., Fan P.F. (2020). Conservation status of otters in China and a discussion on restoring otter populations in the Pearl River Delta. Acta Theriol. Sin..

[75] Smith A., Xie Y. (2009). A Guide to the Mammals of China.

[76] Khoo M., Basak S., Sivasothi N., de Silva P.K., Reza Lubis I. (2021). *Lutrogale* *perspicillata*. IUCN Red List. Threat. Species.

[77] Li F., Chan B. (2017). Past and present: The status and distribution of otters (Carnivora: Lutrinae) in China. Oryx.

[78] Liu Z.X., Sheng H.L. (1998). A jungle cat (*Felis chaus*) discovered in Gansu Province. Acta Theriol. Sin..

[79] Smith A.T., Johnston C.H. (2019). *Lepus* *mandshuricus*. IUCN Red List. Threat. Species.

[80] Shen Y., Liu M., Wang D., Shen X., Li S. (2021). Using an integrative mapping approach to identify the distribution range and conservation needs of a large threatened mammal, the Asiatic black bear, in China. Glob. Ecol. Conserv..

[81] Wang S., Xie Y. (2004). China Species Red List.

[82] Harper L.R., Handley L.L., Carpenter A.I., Ghazali M., Muri C.D., Macgregor C.J., Logan T.W., Law A., Breithaupt T., Read D.S. (2019). Environmental DNA (eDNA) metabarcoding of pond water as a tool to survey conservation and management priority mammals. Biol. Conserv..

[83] Ji Y.Q., Baker C.C.M., Popescu V.D., Wang J.X., Wu C.Y., Wang Z.Y., Li Y.H., Wang L., Hua C.L., Yang Z.X. (2022). Measuring protected-area effectiveness using vertebrate distributions from leech iDNA. Nat. Commun..

[84] de Vries M., Land-Zandstra A., Smeets I. (2019). Citizen scientists’ preferences for communication of scientific output: A literature review. Citiz. Sci. Theory Pract..

[85] China Bird Watching Organization Action Platform (Kunming Vermilion Bird Research Institute) (2021). Annual Report on Bird Observations in China.

[86] Luo J., Li J. (2021). A Review of projects and case studies of citizen science abroad. Stud. Sci. Pop..

[87] Shanghai Forestry General Station (2024). Nine hundred volunteers over three years mapped the distribution of wild raccoons, a successful attempt at citizen science. China Ecol. Civiliz..

[88] IUCN Standard and Petitions Committee (2024). *Guidelines for Using the IUCN Red List Categories and Criteria. Version 16*. Prepared by the Standards and Petitions Committee. https://www.iucnredlist.org/documents/RedListGuidelines.pdf.

[89] Jiang Z.C., Ren Z.T., Zeng X.W., Duan H.X., Wu X.C. (2024). Distribution patterns of national key protected terrestrial wildlife in Yunnan. J. Southwest For. Univ. (Nat. Sci.).

[90] Gaston K.J. (2020). Global patterns in biodiversity. Nature.

[91] Stevens G.C. (1989). The latitudinal gradient in geographical range: How so many species coexist in the tropics. Am. Nat..

[92] Kerr J.T., Packer L. (1997). Habitat heterogeneity as a determinant of mammal species richness in high-energy regions. Nature.

[93] Chi Y., Wang J., Xi C., Qian T., Sheng C. (2020). Spatial pattern of species richness among terrestrial mammals in China. Diversity.

[94] Zhao S., Fang J., Peng C., Tang Z. (2006). The relationships between terrestrial vertebrate species richness in China’s nature reserves and environmental variables. Can. J. Zool..

[95] Norris K., Terry A., Hansford J.P., Turvey S.T. (2020). Biodiversity conservation and the Earth system: Mind the gap. Trends Ecol. Evol..

[96] Mittermeier R.A., Mittermeier C.G., Brooks T.M., Pilgrim J.D., Konstant W.R., da Fonseca G.A.B., Kormos C. (2003). Wilderness and biodiversity conservation. Proc. Natl. Acad. Sci. USA.

[97] Laguardia A., Kamler J.F., Li S., Zhang C., Zhou Z., Shi K. (2017). The current distribution and status of leopards *Panthera pardus* in China. Oryx.

[98] Wang L., Ma Y., Zhou Q., Zhang Y., Savolaimen P., Wang G. (2016). The geographical distribution of grey wolves (*Canis lupus*) in China: A systematic review. Zool. Res..

[99] Catalan J., Ninot J., Aniz M. (2017). High Mountain Conservation in a Changing World.

[100] Beschta R.L., Ripple W.J. (2009). Large predators and trophic cascades in terrestrial ecosystems of the western United States. Biol. Conserv..

[101] Dickinson J.L., Zuckerberg B., Bonter D.N. (2010). Citizen science as an ecological research tool: Challenges and benefits. Annu. Rev. Ecol. Evol. Syst..

[102] McShea W., Shen X.L., Liu F., Wang T.M., Xiao Z.S., Li S. (2020). China’s wildlife camera-trap monitoring needs a unified standard. Biodivers. Sci..

[103] Vitkalova A.V., Feng L.M., Rybin A.N., Gerber B.D., Miquelle D.G., Wang T.M., Yang H.T., Shevtsova E.I., Aramilev V.V., Ge J.P. (2018). Transboundary cooperation improves endangered species monitoring and conservation actions: A case study of the global population of Amur leopards. Conserv. Lett..

[104] Li J., Fu J., Guo X., Zhang Z.H., Li W.Y., Bao Y.N., Ma S.T., Wang Y.C., Gao J. (2021). The potential of cross-border cooperation in border protected areas between China and neighboring countries. J. Nat. Resour..

[105] Ford A.T., Goheen J.R., Augustine D.J., Kinnaird M.F., O’Brien T.G., Palmer T.M., Pringle R.M., Woodroffe R. (2015). Recovery of African wild dogs suppresses prey but does not trigger a trophic cascade. Ecology.

[106] Perino A., Pereira H.M., Navarro L.M., Fernández N., Bullock J.M., Ceaușu S., Cortés-Avizanda A., van Klink R., Kuemmerle T., Lomba A. (2019). Rewilding complex ecosystems. Science.

