# Supplementary material for: Updating the Species Inventory of Large- and Medium-Sized Mammals in China Based on 2009–2020 Field Observation Records
Source: Animals (Basel). 2024 Nov 24;14(23):3380. doi: 10.3390/ani14233380 (PMC11640322; doi:10.3390/ani14233380)
Supplement: Supplementary file 1 [file animals-14-03380-s001.zip › animals-3279610-supplementary.pdf]

Table S1 List of China's 141 large- and medium-sized mammal species included in this study

Species	Latin Name	National Key Protected level	IUCN Red List level
**Primates**			
**Lorisidae**			
Slow Loris	*Nycticebus bengalensis*	I	EN
**Cercopithecidae**			
Slow Loris	*Macaca arctoides*	I	EN
Stump-tailed Macaque	*Macaca assamensis*	II	VU
Assam Macaque	*Macaca cyclopis*	II	VU
Taiwan Macaque	*Macaca leonina*	I	EN
North Pig-tailed Macaque	*Macaca leucogenys*	I	CR
White-cheeked Macaque	*Macaca mulatta*	II	CR
Rhesus Macaque	*Macaca munzala*	II	LC
Arunachal Macaque	*Macaca thibetana*	II	EN
Tibetan Macaque	*Semnopithecus schistaceus*	II	VU
Nepal Gray Langur	*Trachypithecus crepusculus*	I	CR
Indochinese Gray Langur	*Trachypithecus francoisi*	I	EN
François' Langur	*Trachypithecus melamera*	I	EN
Shan State Langur	*Trachypithecus pileatus*	I	VU
Capped Langur	*Trachypithecus leucocephalus*	I	EN
White-headed Langur	*Trachypithecus shortridgei*	I	CR
Shortridgei's Langur	*Rhinopithecus bieti*	I	EN
Yunnan Snub-nosed Monkey	*Rhinopithecus brelichi*	I	EN
Guizhou Snub-nosed Monkey	*Rhinopithecus roxellana*	I	CR
Sichuan Snub-nosed Monkey	*Rhinopithecus strykeri*	I	NT
Myanmar Snub-nosed Monkey	*Rhinopithecus strykeri*	I	CR
**Hylobatidae**			
White-handed Gibbon	*Hylobates lar*	I	CR

Species	Latin Name	National Key Protected level	IUCN Red List level
Western Hoolock Gibbon	*Hoolock hoolock*	I	CR
Gaoligong Hoolock Gibbon	*Hoolock tianxing*	I	CR
Western Black Crested Gibbon	*Nomascus concolor*	I	CR
Eastern Black Crested Gibbon	*Nomascus nasutus*	I	CR
Hainan Gibbon	*Nomascus hainanus*	I	CR
Northern White Cheeked Gibbon	*Nomascus leucogenys*	I	CR
**Pholidota**			
**Manidae**			
Sunda Pangolin	*Manis javanica*	I	CR
Chinese Pangolin	*Manis pentadactyla*	I	CR
**Carnivora**			
**Canidae**			
Golden Jackal	*Canis aureus*	II	DD
Gray Wolf	*Canis lupus*	II	NT
Bengal Fox	*Vulpes bengalensis*	-	DD
Corsac Fox	*Vulpes corsac*	II	NT
Tibetan Fox	*Vulpes ferrilata*	II	NT
Red Fox	*Vulpes vulpes*	II	NT
Racoon Dog	*Nyctereutes procyonoides*	II	NT
Dhole	*Cuon alpinus*	I	EN
**Ursidae**			
Sloth Bear	*Melursus ursinus*	II	EN
Brown Bear	*Ursus arctos*	II	VU
Asiatic Black Bear	*Ursus thibetanus*	II	VU
Sun Bear	*Helarctos malayanus*	I	CR
Giant Panda	*Ailuropoda melanoleuca*	I	VU
**Ailuridae**			
Himalayan Red Panda	*Ailurus fulgens*	II	VU
Chinese Red Panda	*Ailurus styani*	II	VU
**Mustelidae**			
Yellow-throated Marten	*Martes flavigula*	II	VU
Stone Marten	*Martes foina*	II	EN
Sable	*Martes zibellina*	I	VU

Species	Latin Name	National Key Protected level	IUCN Red List level
Wolverine	*Gulo gulo*	I	EN
Steppe Polecat	*Mustela eversmanii*	-	VU
Stripe-backed Weasel	*Mustela strigidorsa*	-	EN
Burmese Ferret-badger	*Melogale personata*	-	EN
Asian Badger	*Meles leucurus*	-	NT
Hog Badger	*Arctonyx collaris*	-	NT
Eurasian Otter	*Lutra lutra*	II	EN
Smooth-coated Otter	*Lutrogale perspicillata*	II	CR
Oriental Small-clawed Otter	*Aonyx cinerea*	II	CR
**Viverridae**			
Large-spotted Civet	*Viverra megaspila*	I	CR
Large Indian Civet	*Viverra zibetha*	I	CR
Small Indian Civet	*Viverricula indica*	I	NT
Asian Palm Civet	*Paradoxurus hermaphroditus*	II	EN
Masked Palm Civet	*Paguma larvata*	-	NT
Binturong	*Arctictis binturong*	I	CR
Small-toothed Palm Civet	*Arctogalidia trivirgata*	I	CR
Owston's Palm Civet	*Chrotogale owstoni*	I	CR
**Prionodontidae**			
Spotted Linsang	*Prionodon pardicolor*	II	VU
**Herpestidae**			
Crab-eating Mongoose	*Herpestes urva*	-	VU
**Felidae**			
Chinese Mountain Cat	*Felis bieti*	I	CR
Jungle Cat	*Felis chaus*	I	CR
Wild Cat	*Felis silvestris*	II	EN
Pallas's Cat	*Otocolobus manul*	II	EN
Leopard Cat	*Prionailurus bengalensis*	II	VU
Eurasian Lynx	*Lynx lynx*	II	EN
Marbled Cat	*Pardofelis marmorata*	II	EN
Asiatic Golden Cat	*Catopuma temminckii*	I	EN
Clouded Leopard	*Neofelis nebulosa*	I	CR
Leopard	*Panthera pardus*	I	EN
Tiger	*Panthera tigris*	I	CR
Snow Leopard	*Panthera uncia*	I	EN
**Proboscidea**			

Species	Latin Name	National Key Protected level	IUCN Red List level
**Elephantidae**			
Asian Elephant	*Elephas maximus*	I	CR
**Perissodactyla**			
**Equidae**			
Przewalski's Horse	*Equus ferus*	I	EW
Asiatic Wild Ass	*Equus hemionus*	I	VU
Tibetan Wild Ass	*Equus kiang*	I	NT
**Cetartiodactyla**			
**Suidae**			
Wild Boar	*Sus scrofa*	-	LC
**Camelidae**			
Bactrian Camel	*Camelus ferus*	I	CR
**Tragulidae**			
Lesser Mouse Deer	*Tragulus kanchil*	I	EN
**Moschidae**			
Anhui Musk Deer	*Moschus anhuiensis*	I	EN
Forest Musk Deer	*Moschus berezovskii*	I	CR
Alpine Musk Deer	*Moschus chrysogaster*	I	CR
Black Musk Deer	*Moschus fuscus*	I	CR
Himalayan Musk Deer	*Moschus leucogaster*	I	EN
Siberian Musk Deer	*Moschus moschiferus*	I	CR
**Cervidae**			
Chinese Water Deer	*Hydropotes inermis*	II	VU
Tufted Deer	*Elaphodus cephalophus*	II	NT
Black Muntjac	*Muntiacus crinifrons*	I	EN
Fea's Muntjac	*Muntiacus feae*	-	DD
Gongshan Muntjac	*Muntiacus gongshanensis*	II	EN
Reeves' Muntjac	*Muntiacus reevesi*	-	NT
Red Muntjac	*Muntiacus vaginalis*	-	NT
Eld's Deer	*Rucervus eldii*	I	CR
Sambar	*Rusa unicolor*	II	NT
Sika	*Cervus nippon*	I	EN
White-lipped Deer	*Przewalskium albirostris*	I	EN
Red Deer	*Cervus elaphus*	I	CR
Père David's Deer	*Elaphurus davidianus*	I	CR
Siberian Roe Deer	*Capreolus pygargus*	-	NT
Moose	*Alces alces*	I	CR
**Bovidae**			
Gaur	*Bos gaurus*	I	CR
Wild Yak	*Bos mutus*	I	VU

Species	Latin Name	National Key Protected level	IUCN Red List level
Mongolian Gazelle	*Procapra gutturosa*	I	CR
Tibetan Gazelle	*Procapra picticaudata*	II	NT
Przewalski's Gazelle	*Procapra przewalskii*	I	EN
Goitered Gazelle	*Gazella subgutturosa*	II	VU
Tibetan Antelope	*Pantholops hodgsonii*	I	NT
Chinese Takin	*Budorcas tibetana*	I	VU
Himalayan Takin	*Budorcas taxicolor*	I	CR
Red Goral	*Naemorhedus baileyi*	I	EN
Long-tailed Goral	*Naemorhedus caudatus*	II	CR
Burmese Goral	*Naemorhedus evansi*	II	DD
Himalayan Goral	*Naemorhedus goral*	I	EN
Chinese Goral	*Naemorhedus griseus*	II	VU
Himalayan Tahr	*Hemitragus jemlahicus*	I	CR
Siberian Ibex	*Capra sibirica*	II	NT
Bharal	*Pseudois nayaur*	II	LC
Argali	*Ovis ammon*	I	CR
Chinese Serow	*Capricornis milneedwardsii*	II	VU
Taiwan Serow	*Capricornis swinhoei*	I	NT
Himalayan Serow	*Capricornis thar*	I	EN
Red Serow	*Capricornis rubidus*	II	DD
**Rodentia**			
**Castoridae**			
Eurasian Beaver	*Castor fiber*	I	CR
**Hystricidae**			
Asiatic Brush-tailed Porcupine	*Atherurus macrourus*	-	LC
Malayan Porcupine	*Hystrix brachyura*	-	LC
**Lagomorpha**			
**Leporidae**			
Yunnan Hare	*Lepus comus*	-	NT
Korean Hare	*Lepus coreanus*	-	LC
Hainan Hare	*Lepus hainanus*	II	CR
Manchurian Hare	*Lepus mandshuricus*	-	LC
Woolly Hare	*Lepus oiostolus*	-	LC
Chinese Hare	*Lepus sinensis*	-	LC
Desert Hare	*Lepus tibetanus*	-	LC
Mountain Hare	*Lepus timidus*	II	LC
Tolai Hare	*Lepus tolai*	-	LC

Species	Latin Name	National Key Protected level	IUCN Red List level
Yarkand Hare	*Lepus yarkandensis*	II	NT

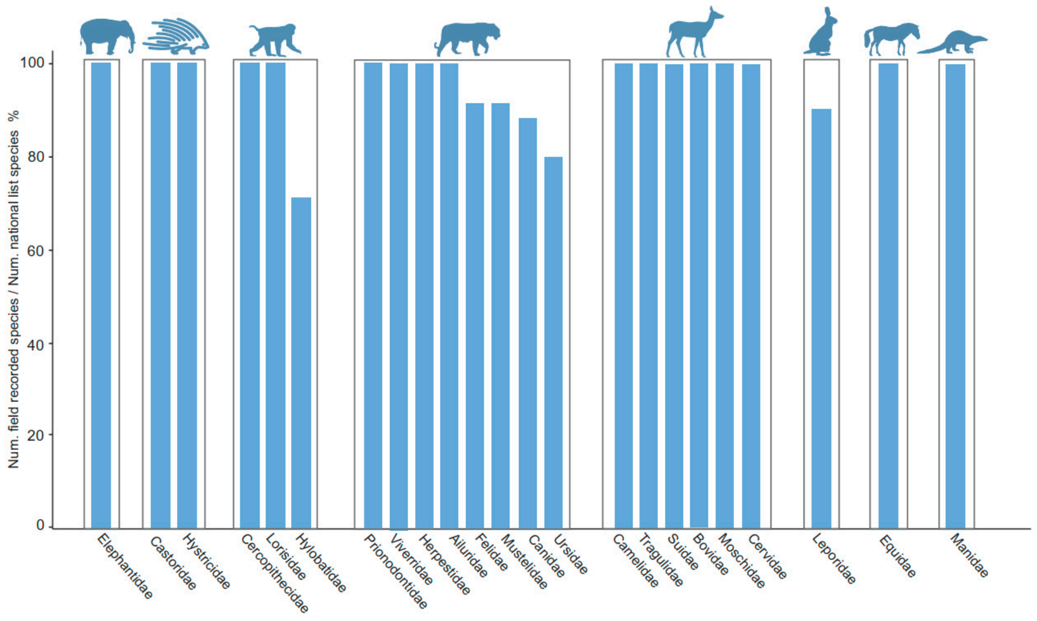

Figure S1. The percentage of recorded large- and medium-sized mammal species compared to the national species list in each family.

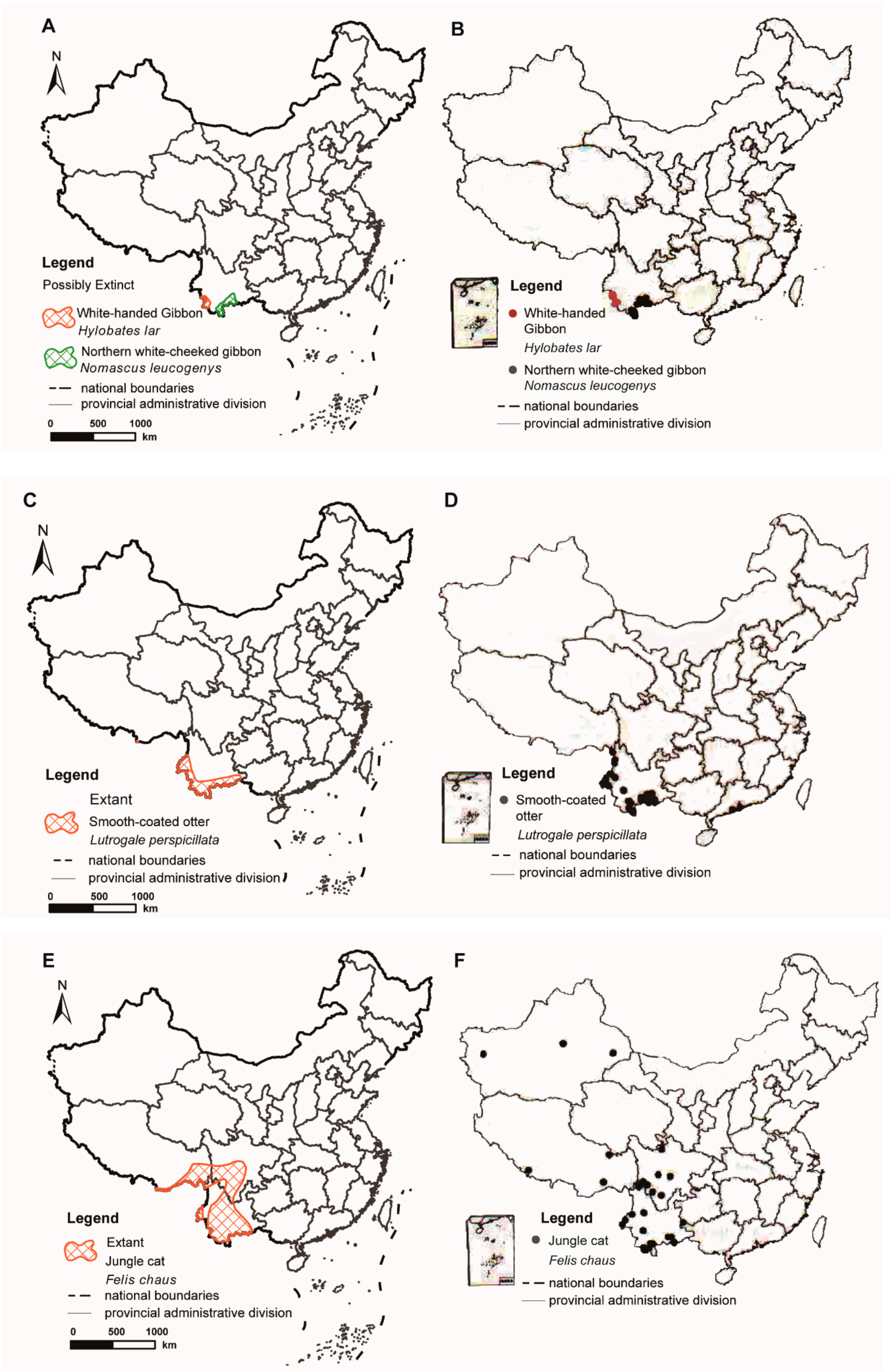
A
N
Legend
Possibly Extinct
White-handed Gibbon
Hylobates lar
Northern white-cheeked gibbon
Nomascus leucogenys
national boundaries
provincial administrative division
0 500 1000
km
B
Legend
White-handed Gibbon
Hylobates lar
Northern white-cheeked gibbon
Nomascus leucogenys
national boundaries
provincial administrative division
C
N
Legend
Extant
Smooth-coated otter
Lutrogale perspicillata
national boundaries
provincial administrative division
0 500 1000
km
D
Legend
Smooth-coated otter
Lutrogale perspicillata
national boundaries
provincial administrative division
E
N
Legend
Extant
Jungle cat
Felis chaus
national boundaries
provincial administrative division
0 500 1000
km
F
Legend
Jungle cat
Felis chaus
national boundaries
provincial administrative division

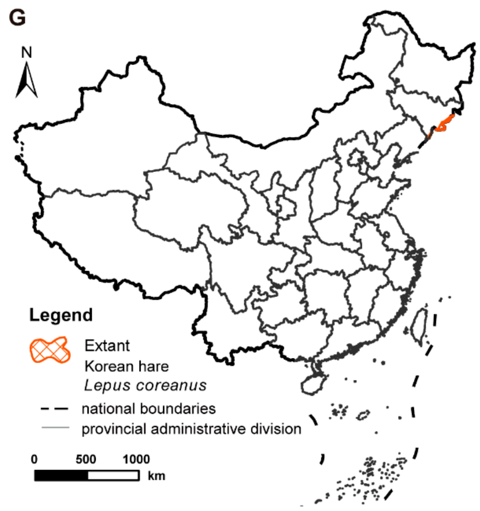

Figure S2. The distribution of large- and medium-sized mammals in China that are not recorded in IUCN (left, polygon) and the China Species Red List (right, point). Figure A-G shows the distribution of species from IUCN on the left and the record location given by the species in the China species red list on the right. Figure G species has only IUCN source distribution map, no China species red list source distribution.
